# External Physical Demands during Official Under-18 Basketball Games: Consideration of Overtime Periods

**DOI:** 10.5114/jhk/185682

**Published:** 2024-07-17

**Authors:** Carlos Sosa-Marin, Enrique Alonso-Pérez-Chao, Juan Trapero, Carlos Ribas, Anthony S. Leicht, Alberto Lorenzo, Sergio L. Jiménez

**Affiliations:** 1 Faculty of Sport Sciences, University Polytechnic of Madrid, Madrid, Spain.; 2 Faculty of Sport Sciences, European University of Madrid, Madrid, Spain.; 3 Faculty of Sports Sciences, University Alfonso X el Sabio, Community of Madrid, Spain.; 4 Sport and Exercise Science, James Cook University, Townsville, Australia.; 5 Sport Sciences Research Centre, Universidad Rey Juan Carlos, Madrid, Spain.

**Keywords:** team sports, microtechnology, local positioning system, fatigue, basketball demands

## Abstract

The purpose of this study was to identify the external demands during official under-18 basketball matches that included four quarters (Q1, Q2, Q3, Q4) and/or overtime (OT) periods. Variables included: 1) distance covered and distance covered within four intensity zones (standing-walking S-W, jogging JOG, running RUN, and high-speed running HSR); 2) explosive efforts per minute (EEs); 3) PlayerLoad (PL); 4) the number of jumps per minute; 5) the number of accelerations above 2 m·s2 (ACCs); and 6) the number of decelerations above −2 m·s2 per minute (DECs). The values for total distance, JOG, RUN, HSR, PL, ACC, and DEC were significantly smaller during periods Q2, Q3, Q4, and OT compared to Q1. In addition, the number of EEs during Q1 was significantly greater than during Q3, Q4, and OT. When comparing OT periods to Q2, there were significantly smaller values for total distance, RUN, and PL. Similarly, when comparing OT periods to Q3, significantly smaller values were found for PL. Furthermore, when comparing OT periods to Q4, significantly smaller values were achieved for total distance and PL. These findings indicate that players experienced a decrease in external physical demands as the match progressed to an OT period. This decrease may be indicative of player fatigue and/or strategic changes that should be considered by practitioners in the preparation and management of players for overall team success in junior basketball competitions.

## Introduction

One of the first methods used to document basketball player’s movements was the cartographic technique, a time-consuming and limited approach (Hulka et al., 2014). Since then, many studies have quantified the movements or external activity demands of basketball players during matches, predominantly using time-motion analysis (TMA) (Matthew and Delextrat, 2009; Scanlan et al., 2015). However, TMA has limitations (Buchheit et al., 2014) with more time effective, valid, and reliable technologies available to monitor player movements within indoor sports. For example, Electronic Performance Tracking Systems (EPTS) including inertial movement units (IMUs) or accelerometers, gyroscopes, and magnetometers (Chambers et al., 2015; Gabbett, 2013) and local positioning systems (LPS) (Serpiello et al., 2018) have become standard monitoring tools for basketball practitioners (Fox et al., 2020a; Portes et al., 2019). Important advantages of wearable/EPTS technology include the capability to monitor several players simultaneously in real time that allows immediate and cost-effective analyses of movement patterns (Aughey and Falloon, 2010). Despite the availability and benefits of this technology to monitor players’ external demands, such wearable devices are normally prohibited during professional basketball matches by many competitions/leagues. Subsequently, basketball match-play demands using EPTS have been predominantly documented in junior players such as those under-18 years of age (U-18) (Alonso Pérez-Chao et al., 2022; Sosa et al., 2021). Data derived from U-18 basketball studies indicate that basketball players engage in an intermittent, high-intensity sport in which they spend most of their match time (93.7%) standing-walking (<7 km·h^−1^) and jogging (7–14 km·h^−1^) (Sosa et al., 2021). These low intensity aerobic activities are interspersed with high-intensity movements involving continuous changes of direction, jumps, accelerations (ACCs), decelerations (DECs), and specific basketball skills (e.g., crossover, lay-ups) (Alonso et al., 2020; Alonso Pérez-Chao et al., 2023; Stojanović et al., 2018).

While a variety of match movements and external demands have been documented for U-18 basketball players (Alonso et al., 2020; Vázquez-Guerrero et al., 2019a), a unique match response was observed with players experiencing a decrease in distance covered, overall demands or effort (PlayerLoad), the number of high intensity ACCs and DECs between the first and the last quarter of matches (Vázquez-Guerrero et al., 2019b). Similar responses were observed for the most demanding stages of U-18 male basketball matches with peak intensities (Alonso Pérez-Chao et al., 2022; Fox et al., 2020b), specifically for running-based demands (e.g., distance, PlayerLoad, and high-speed running) (Alonso Pérez-Chao et al., 2022) decreasing between the first and last quarters of matches (Alonso Pérez-Chao et al., 2022). These reductions in match external demands could be related to: 1) strategic changes in the playing intensity or style; 2) situational influences such as more stoppages (e.g., a greater number of fouls committed) and consequently longer quarter/match duration; or 3) accumulated player fatigue (Alonso Pérez-Chao et al., 2022; Bradley and Noakes, 2013; García et al., 2020). While average and peak external demands during standard basketball matches have been examined (Alonso Pérez-Chao et al., 2022; Fox et al., 2020b; Vázquez-Guerrero et al., 2019b), matches can also include extra or overtime (OT) periods where the scores are tied at the end of matches and further play is needed to identify a winner. This extra match time will add to the external demands experienced by players with examination of matches that include scant OT periods. Investigation of such matches including OT periods may provide a more comprehensive understanding of players’ external demands needed to play in any match likely to be experienced during a competition season. Furthermore, an understanding of the fluctuations in these external physical requirements would allow basketball practitioners to develop more precise conditioning practices, optimize player’s performance across specific match periods, and develop strategies for managing fatigue during matches. Therefore, the aim of this study was to document the external demands (via microtechnology/LPS) encountered by players across quarters and/or OT periods during official U-18 matches. Based on prior reports (Alonso et al., 2020; Portes et al., 2022), it was hypothesized that the average external physical demands would decline throughout the match with the smallest values occurring during the OT period.

## Methods

### Participants

Elite, male basketball players from the same team (n = 12, mean ± standard deviation [SD]: age: 16.9 ± 0.8 years, body height: 196.0 ± 9.5 cm, body mass: 91.8 ± 8.3 kg) were monitored during twenty-three official matches played within the same gym. Match recordings from each player were retained for final analysis if they consisted of a minimum of 15 minutes of playing time per match (Alonso Perez-Chao et al., 2021, 2023) for at least 35% of the total number of matches (n = 8). Playing time was classified as the time (minutes) each player was on the court during each quarter, OT periods and the match including stoppages (i.e., free-throws, fouls, out-of-bounds, rule infringements), but excluding the warm-up, breaks between quarters and time-outs. Initially, 15 players volunteered for this study, but recordings from three players did not meet the above eligibility criteria and were excluded from the final analysis. Overall, 550 recordings (quarters/OT) from 12 players were obtained and included in analyses.

### Measures

To account for different match playing times for players, all variables were normalized to the number of minutes played by each player during the match (Coyne et al., 2018). The variables recorded were: total distance (m·min^−1^) covered by players while on the court and distance (m·min^-1^) covered within four intensity zones including standing-walking [S-W] = <7 km·h^−1^, jogging [JOG] = 7–14 km·h^−1^, running [RUN] = 14.01–18 km·h^−1^, and high-speed running [HSR] >18 km·h^−1^, as previously reported for basketball athletes (Sosa et al., 2021); explosive efforts (EEs, number·min^−1^); PlayerLoad (PL, absolute units – AU·min^−1^); the number of jumps >20 cm (number·min^−1^); the number of accelerations (ACCs) above 2 m·s^2^ (number·min^−1^); and the number of decelerations (DECs) below −2 m·s^2^ (number·min^−1^).

Explosive efforts were derived from the inertial movement analyses (IMA) and considered all ACCs, DECs and changes of directions (Svilar et al., 2018). PlayerLoad was calculated as the square root of the sum of the instantaneous rate of change in ACCs within the three movement planes (x-, y-, and z-axis) using the following formula (Brown and Greig, 2017): PlayerLoad™ = [√((⟦fwd⟧_(t=i+1)-⟦fwd⟧_(t=i) )⟦2 )+√(⟦side⟧_(t=i+1)-⟦side⟧_(t=i) )⟦2+√(⟦up⟧_(t=i+1)-⟦up⟧_(t=i) )⟦2]/100, where fwd indicated movement in the anterior-posterior direction, side indicated movement in the medial-lateral direction, up indicated vertical movement, and t represented time. The dwell times used for ACC and DEC calculations were set as 0.3 s given values between 0.3 and 0.4 s have been typically used in prior basketball studies (Alonso Perez-Chao et al., 2021, 2022, 2023; Svilar et al., 2018).

### Design and Procedures

This observational investigation was performed across a 9-month period throughout the 2019–2020 season where match-play was conducted in accordance with official FIBA rules (i.e., 4 x 10-min quarters, 5-min OT periods) and officiated by experienced and qualified referees. Each player wore a device (Vector S7; Catapult Sports, Melbourne, Australia) that was incorporated into a fitted neoprene vest under their regular playing uniform and positioned on the upper thoracic spine between the scapulae (Hodder et al., 2020). Each device contained microsensor technology consisting of an accelerometer (±16 g, 100 Hz), a magnetometer (±4.900 µT, 100 Hz), and a gyroscope (up to 2,000 deg/s, 100 Hz). Each device also contained a LPS transmitter sampling at 10 Hz. The LPS utilized for each match was an ultra-wide band, a -GHz transmitting system equipped with 24 anchors positioned around the perimeter of the court/stadium. The technology used in this study has been reported to be valid in measuring distance (Hodder et al., 2020; Hoppe et al., 2018; Luteberget et al., 2018; Serpiello et al., 2018), speed, accelerations, decelerations (Hodder et al., 2020; Luteberget et al., 2018), and PlayerLoad (Luteberget et al., 2017). All players were familiarized with the monitoring technology, having worn the devices during training sessions and matches in the previous season. Each device was turned on ~20–40 min before the warm-up preceding each match. Players wore the same device throughout the study period to avoid inter-device variation in external demand output (Johnston et al., 2014).

### Statistical Analysis

All values are presented as means, SD and CV for each period of the match as follows: quarter 1 (Q1), quarter 2 (Q2), quarter 3 (Q3), quarter 4 (Q4) and OT periods. The linear mixed model (LMM) for repeated measures was used to identify differences between periods for variables. Within each LMM, the ‘period’ was the fixed effect while 'Player’ was the random effect. Periods (Q1, Q2, Q3, Q4 and OT) were included as nominal predictor variables in the LMM. Estimated magnitude of difference in means (i.e., effect size, ES) and their 95% confidence limits were presented in standardized units and evaluated qualitatively as follows: trivial, 0–0.2; small, 0.2–0.6; moderate, 0.6–1.2; large, 1.2–2.0; and very large, >2.0 (Hopkins et al., 2009). Descriptive analysis, LMM and post-hoc pairwise comparisons with Bonferroni correction were conducted using a standard statistical software package (IBM SPSS for Windows, version 23, IBM Corporation, Armonk, New York) while ES was calculated using a customized Microsoft Excel spreadsheet (version 16.0, Microsoft Corporation, Redmond, WA).

## Results

Descriptive statistics (mean ± SD and CV) for each variable during the match periods are presented in Table 1.

**Table 1:** Descriptive statistics of the external physical demand variables during each match period.

		Q1	Q2	Q3	Q4	OT
**Total distance (m/min)**	Mean ± SD	90.9 ± 11.3	80.3 ± 10.7^a^	81.0 ± 11.6^a^	79.6 ± 10.8^a^	71.0 ± 11.4^abd^
	%CV	12.5%	13.3%	14.4%	13.6%	16.1%
**S-W distance (m/min)**	Mean ± SD	35.5 ± 3.5	35.8 ± 4.3	36.9 ± 5.5	36.2 ± 4.2	34.4 ± 3.1
	%CV	10.0%	12.1%	15.1%	11.8%	9.2%
**JOG distance (m/min)**	Mean ± SD	34.7 ± 7.0	28.8 ± 6.4^a^	29.1 ± 9.0^a^	29.0 ± 6.6^a^	26.3 ± 7.4^a^
	%CV	20.3%	22.3%	31.1%	23.0%	28.5%
**RUN distance (m/min)**	Mean ± SD	14.6 ± 4.8	10.9 ± 3.8^a^	10.7 ± 4.4^a^	10.4 ± 3.8^a^	7.7 ± 3.8^a^
	%CV	32.9%	34.9%	41.9%	36.9%	50.2%
**HSR distance (m/min)**	Mean ± SD	6.0 ± 3.1	4.6 ± 2.8^a^	4.2 ± 2.5^a^	3.9 ± 2.1^a^	2.6 ± 1.5^a^
	%CV	52.4%	62.5%	59.9%	55.2%	57.8%
**EE (number/min)**	Mean ± SD	3.7 ± 1.3	3.3 ± 1.1	3.3 ± 1.1^a^	3.2 ± 1.1^a^	2.6 ± 1.2^a^
	%CV	35.7%	32.6%	34.4%	34.2%	45.4%
**PL (AU/min)**	Mean ± SD	10.3 ± 2.4	9.0 ± 1.7^a^	9.2 ± 1.9^a^	9.0 ± 1.8^a^	7.3 ± 1.4^abcd^
	%CV	24.0%	18.9%	21.1%	20.7%	19.6%
**Jumps (number/min)**	Mean ± SD	0.7 ± 0.3	0.6 ± 0.3	0.7 ± 0.3	0.6 ± 0.2	0.5 ± 0.3
	%CV	53.5%	50.7%	50.2%	43.4%	56.3%
**ACC (number/min)**	Mean ± SD	3.4 ± 0.8	2.8 ± 0.9^a^	2.7 ± 0.9^a^	2.7 ± 0.8^a^	2.4 ± 0.6^a^
	%CV	24.6%	34.3%	35.3%	30.7%	25.7%
**DEC (number/min)**	Mean ± SD	2.9 ± 0.9	2.5 ± 0.9^a^	2.3 ± 0.9^a^	2.3 ± 0.8^a^	2.2 ± 0.7^a^
	%CV	30.3%	36.9%	39.0%	35.3%	33.0%

Abbreviations: S-W = standing-walking, JOG = jogging, RUN = running, HSR = high speed running, EE = explosive efforts, PL = player load, ACC = acceleration, DEC = decelerations, Q1 = quarter 1, Q2 = quarter 2, Q3= quarter 3 and Q4 = quarter 4. Notes: ^a^ p < 0.05 vs. Q1, ^b^ p < 0.05 vs. Q2, ^c^ p < 0.05 vs. Q3, ^d^ p < 0.05 vs. Q4

Compared to Q1, significantly smaller values were achieved during Q2, Q3, Q4 and OT periods for total distance (*p* < 0.001), JOG distance (*p* < 0.001), RUN distance (*p* < 0.001), HSR distance (*p* < 0.001), PL (*p* < 0.001), ACCs (*p* < 0.001) and DECs (Q2, *p* = 0.001; Q3 and Q4, *p* < 0.001; OT, *p* = 0.029). Furthermore, EE values during Q1 were significantly greater compared to Q3 (*p* = 0.023), Q4 (*p* = 0.022) and OT (*p* = 0.007). Compared to Q2, significantly smaller values were achieved during OT for total distance (*p* = 0.018), RUN distance (*p* = 0.038) and PL (*p* = 0.009). Compared to Q3, significantly smaller values were achieved during OT for PL (*p* = 0.002) only. Compared to Q4, significantly smaller values were achieved during OT for total distance (*p* = 0.040) and PL (*p* = 0.015). No other significant (*p* > 0.05) differences between periods were apparent for variables.

**Figure 1:**
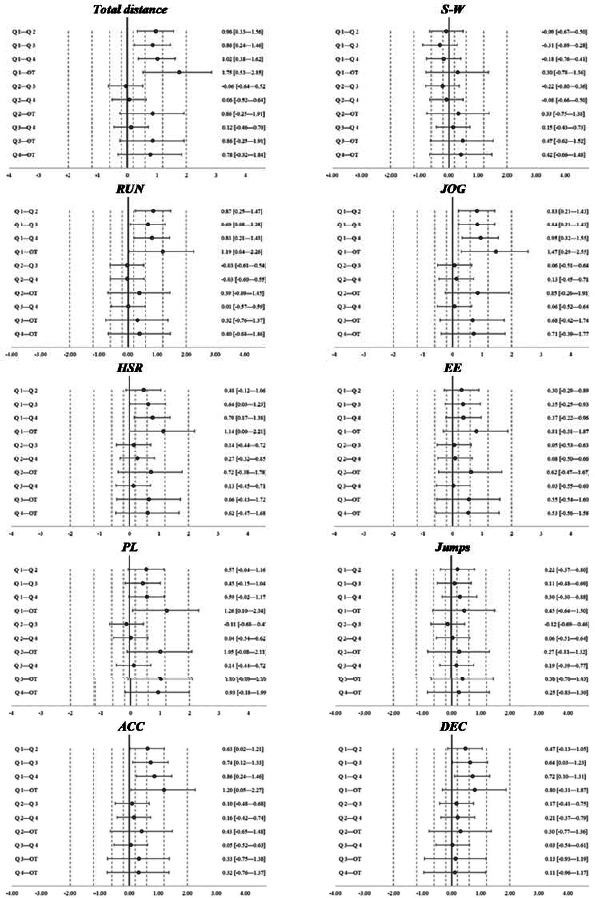
Standardized effect size magnitude [95% confidence interval] between periods for all variables. ***Notes:*** The dotted lines represent the effect size magnitude thresholds from trivial to large (see Methods). ***Notes:*** *variables shown are: total distance (m·min^−1^), distance (m·min^−1^) covered within four intensity zones including standing-walking [S-W] = <7 km·h^−1,^ jogging [JOG] = 7–14 km·h^−1^, running [RUN] = 14.01–18 km·h^−1,^, and high-speed running [HSR] >18 km·h^−1^, explosive efforts (EEs, number·min^−1^); PlayerLoad (AU·min^−1^); the number of jumps >20 cm (number·min^−1^); the number of accelerations (ACC) above 2 m·s^2^ (number·min^−1^); and the number of decelerations (DEC) below −2 m·s^2^ (number·min^−1^)*

The effect size of differences between periods was mostly trivial to moderate in magnitude (Figure 1). Large ES was noted for comparisons between Q1 and OT for total distance, RUN distance, PL, and ACCs (Figure 1).

## Discussion

The aim of the study was to examine the external demands (via microtechnology/LPS) encountered by players during official U-18 matches involving OT periods. There were several novel findings that support a better understanding of the fluctuations in average external physical demands during matches. In this regard, players experienced: 1) greater Q1 values for total distance, JOG, RUN, HSR, EE, PL, ACCs, and DECs compared to other periods; 2) smaller OT values for PL and total distance compared to other periods; and 3) no significant changes in jumps or S-W distance between any period of the match. These results support our hypothesis that the average external physical demands for U-18 basketball players decline across matches with lower values during OT. These results also provide useful insights for basketball practitioners when designing match-specific training (e.g., inclusion of endurance drills when fatigued) and tactical strategies (e.g., recovery strategies such as nutrition during half-time) to maximize team success.

The average external physical demands decreased throughout the U-18 match with Q4 and OT being the periods with the smallest values of total distance, JOG, RUN and HSR. The decline in these locomotion-based demands was most notable during the OT period and may be associated with situation-related or strategic factors such as altered game pace and/or stoppages in play due to time-outs and free-throws following commitment of fouls to prolong the match (Alonso Perez-Chao et al., 2021, 2022). Furthermore, the decline in external physical demands may be due to fatigue as a result of muscle damage, a decrease in glycogen stores, and increased self-perception of fatigue (Gibson and Noakes, 2004; Noakes et al., 2005). The current results of lower Q4 values for total distance and HSR compared to Q1 were similar to those previously reported for U-18 male basketball players (Alonso Pérez-Chao et al., 2022; García et al., 2020; Vázquez-Guerrero et al., 2019b). Importantly, the current study extended these results to demonstrate that players experienced even less total distance (*p* = 0.040) and PL (*p* = 0.015) during OT periods compared to Q4. These worse player movements may be associated with fatigue (Gibson and Noakes, 2004; Noakes et al., 2005) or strategic factors (Alonso Perez-Chao et al., 2021, 2022) that require unique attention by coaches and strength and conditioning practitioners. Better prepared players through physical and/or strategic initiatives may be at an advantage to excel during extremely important periods of the match (i.e., Q4, OT). Likewise, less prepared players may be significantly disadvantaged at these match times with team success likely to be poor.

While significant reductions in PL and total distance were noted in the current study, decreases in ACCs and DECs were also observed during matches. Prior studies have also reported significant decreases in ACCs and DECs from Q4 to Q1 in U-18 and U-19 basketball players (Abdelkrim et al., 2007, 2010; Vázquez-Guerrero et al., 2020). Additionally, García et al. (2020) reported that ACCs, DECs and jump differences between Q1 and other match quarters were significant with this difference being the greatest between Q1 and Q4. The reduction in these IMA variables may also support the existence of fatigue later in the match (Gibson and Noakes, 2004; Noakes et al., 2005) or strategic actions to slow the play (Alonso Perez-Chao et al., 2021, 2022; Stojanović et al., 2018). It remains to be seen whether such a strategic/tactical focus was implemented by players to counteract resultant match fatigue or a means for match success, irrespective of player fatigue levels. Future studies examining player and/or coach intent at/or near the end of the match may clarify the origin of such late-match changes in players’ external physical demands and their impact on practitioners’ preparation of players.

This study offers important practical value to basketball practitioners. First, the present findings of reductions in external physical demands across the match including an OT period may help basketball conditioning professionals optimally formulate training plans to enhance the preparation of players to cope with all potential demands likely to be experienced across an entire match. For example, a focus on increasing total distance, RUN, HSR, EEs, PL, ACCs, and DECs during specific endurance drills could enhance players’ fitness resilience to better maintain this type of activity across matches. Second, the present results may assist coaches with players’ recovery and substitution strategies to ensure that physical capacity and performance of players and the team are maintained at optimal levels throughout the match. Consideration of rest time between quarters, nutritional strategies at half-time (e.g., carbohydrate, caffeine, etc.), and second-half substitution patterns by coaches may enhance players’ recovery and maintenance of activity intensity later in the match for the overall team success.

While novel results were identified in the current study, some limitations of this study should be considered when interpreting our results. Firstly, only external physical load variables were monitored with internal load variables (e.g., heart rate) not explored. Future investigations examining concurrent internal loads during matches would provide additional valuable insights for basketball practitioners and coaches when designing match-specific training and tactical strategies. Secondly, a single elite, junior, male basketball team was examined in the current study with further research needed to expand our results to other teams and a wider sample of male and female basketball players of all ages.

## Conclusions

The current study identified that total distance, RUN, HSR, EEs, PL, ACCs, and DECs were significantly reduced during U-18 basketball matches with the lowest values occurring during the latter part of matches (e.g., Q4, OT). Furthermore, total distance and PL were significantly smaller during OT periods compared with Q4, possibly due to fatigue and/or match strategies for slow play. The present findings of reduced match demands over time may assist basketball conditioning professionals in optimally formulating training plans to enhance the preparation of players to cope with the latter match demands. Additionally, the decrease in players’ movements during the latter periods of matches may require coaches to consider rest time between quarters, nutritional strategies and second-half substitution patterns as strategic practices to enhance players’ recovery and performance in the latter part of matches for team success.
